# Psychometric properties of the Post-Traumatic Growth Inventory-Short Form (PTGI-SF) in Chilean population exposed to the February 27th earthquake: analysis through Exploratory Structural Equation Modeling

**DOI:** 10.3389/fpsyg.2026.1642729

**Published:** 2026-05-14

**Authors:** Carlos Serrano, Cristian Cáceres, Marcelo Nvo-Fernández, Susan Galdames, Valentina Miño-Reyes, Marcelo Leiva-Bianchi

**Affiliations:** 1 Faculty of Social Sciences and Humanities, Universidad Autónoma de Chile, Talca, Chile; 2 Universidad Católica del Maule, Facultad de Ciencias de la Salud, Departamento de Psicología, Curicó, Chile; 3 Departamento de Psicología Universidad de La Serena, La Serena, Chile; 4 Independent Researcher, Talca, Chile; 5 Laboratory of Methodology, Behavioural Sciences and Neuroscience, Faculty of Psychology, Universidad de Talca, Talca, Chile

**Keywords:** Chile earthquake, Exploratory Structural Equation Modeling, measurement invariance, natural disasters, post-traumatic growth, psychometric properties

## Abstract

**Introduction:**

The Post-Traumatic Growth Inventory-Short Form (PTGI-SF) has shown substantial cross-cultural variability in its factorial structure, yet contemporary latent-variable validation evidence remains limited for Chilean disaster-exposed populations.

**Methods:**

This study evaluated the psychometric properties of the PTGI-SF in 578 community-dwelling adults from three affected localities (Cauquenes, Pelluhue, and Tongoy) exposed to the February 27, 2010, Chile earthquake. Internal structure was examined using Exploratory Structural Equation Modeling (ESEM), reliability was estimated with McDonald's ordinal omega, and validity evidence was examined via ROC analysis and correlations with posttraumatic stress symptoms assessed using the SPRINT-E. Factorial similarity in loading patterns across localities was described using Tucker's congruence coefficients, whereas measurement equivalence was tested through multigroup CFA measurement invariance (MI) analyses.

**Results:**

ESEM supported a culturally specific three-factor solution (Appreciation of Life, Spiritual Change, and Self-Perception), diverging from the original five-factor model. Reliability was high across localities (Omega = 0.91–0.95). Model fit varied by context, with acceptable fit in Tongoy (RMSEA = 0.08; CFI/TLI = 0.99), mixed-to-poor fit in Pelluhue (RMSEA = 0.14; CFI = 0.98; TLI = 0.95), and clearly unacceptable fit in Cauquenes (RMSEA = 0.22; CFI = 0.92; TLI = 0.80). Discriminative validity relative to severe PTSD symptomatology (SPRINT-E ≥7 intense symptoms) was moderate (AUC = 0.703; sensitivity = 90.91%; specificity = 48.69%), and concurrent validity showed a significant positive association with PTSD symptoms (*r* = 0.288, *p* < 0.001).

**Discussion:**

Measurement invariance analyses supported configural and metric invariance across localities, whereas scalar invariance could not be established due to inadmissible solutions, indicating that latent mean comparisons across communities are not supported.

## 1 Introduction

In the initial phases following a disaster, both the general population and professionals typically focus their attention on the psychological weaknesses that could emerge because of the adverse event. The scientific literature has preferentially described this impact around disruptive responses such as post-traumatic stress symptoms (Xue et al., 2015; García, 2011). According to the psychosocial impact model of trauma, these disruptive responses would be more frequent in vulnerable populations (Leiva-Bianchi et al., 2018), characterized by greater previous exposure to deprivations and adversities. Recent meta-analytic evidence also indicates that specific cumulative and interpersonal adversities substantially increase the risk of developing complex trauma presentations, including complex PTSD (Leiva-Bianchi et al., 2023). This perspective is reflected in that approximately 90% of trauma-related studies have concentrated on negative psychological, physical, or social sequelae (Linley and Joseph, 2004).

However, recent research suggests that the percentage of individuals who develop psychopathologies after traumatic events is lower than those who remain asymptomatic (Roeckner et al., 2021). A review by Cho and Park (2013) indicates that between 55 and 83% of people exposed to traumatic events experience psychological growth. In specific clinical contexts, such as HIV diagnosis, approximately 60% of patients reported stable positive changes (Milam, 2004). Traumatic experiences not only generate distress but can also foster positive cognitive and behavioral changes, termed Post-Traumatic Growth (PTG; Joseph and Linley, 2008; Tedeschi and Calhoun, 1996; Triplett et al., 2012), present even with severe symptoms (Nvo-Fernández et al., 2025).

PTG is defined as the positive psychological change experienced after struggling with traumatic events, manifested in three dimensions: changes in self-perception (recognition of personal strength), improvement in interpersonal relationships (greater intimacy and emotional expression), and transformation in life philosophy (increased appreciation and spiritual change) (Tedeschi and Calhoun, 2004).

Tedeschi and Calhoun (1996) have proposed that PTG manifests in three fundamental dimensions: self-perception, interpersonal relationships, and life philosophy. The self- perception dimension implies significant internal changes, where the individual recognizes their own strength and capacity to face adversities (Tedeschi et al., 2018). On the other hand, changes in interpersonal relationships are usually reflected in the strengthening of social bonds and in greater ability for empathy and emotional expression (Svetina and Nastran, 2012; Tedeschi and Calhoun, 2004). Finally, life philosophy is affected in terms of increased appreciation toward life and greater gratitude (Joseph et al., 1993; Tedeschi et al., 2018).

To evaluate these changes, Tedeschi and Calhoun (1996) developed the Post-Traumatic Growth Inventory (PTGI), composed of 21 items distributed across five dimensions: relating to others, new possibilities, Personal strength, Spiritual change, and Appreciation of life. Although other similar instruments exist such as the Stress-Related Growth Scale (Park et al., 1996) and the Post-Traumatic Changes Questionnaire (Joseph et al., 2012), the PTGI is the most widely used due to its robust psychometric properties and extensive cross-cultural validation (Joseph and Linley, 2008; García et al., 2013; Tedeschi et al., 2018).

However, the factorial structure of the PTGI has been the subject of considerable debate in scientific literature. While the original model by Tedeschi and Calhoun (1996) proposes five factors (Relating to others, new possibilities, Personal strength, Spiritual change, and Appreciation of life), subsequent studies have reported notable structural variability according to cultural context and the population studied. Specifically, three- factor solutions have been identified in Latino and Mediterranean populations (García et al., 2013; Garrido-Hernansaiz et al., 2017), four-factor models in Asian samples (Taku et al., 2008), and unidimensional structures in populations with specific traumas (Sears et al., 2003; Osei-Bonsu et al., 2012). Additionally, controversy persists about the hierarchical nature of the construct: while some studies support a first-order correlated factors model (Cobb et al., 2006; Joseph et al., 2004), others evidence a hierarchical structure with PTG as a second-order factor that encompasses more specific dimensions (Cadell et al., 2003; Morris et al., 2005). This factorial heterogeneity suggests that cultural differences, the type of trauma experienced, and methodological decisions (such as the factorial extraction and rotation method employed) significantly influence the identified structure of PTG (Weiss and Berger, 2010), posing important challenges for cross-cultural validation of the instrument.

This variability could be explained by cultural or methodological differences (Garrido-Hernansaiz et al., 2017; Tomita et al., 2016). Additionally, the length of the original instrument has posed challenges, especially in clinical contexts or emergencies where conditions require briefer and more efficient instruments (Cann et al., 2010).

To address its limitations, Cann et al. (2010) developed an abbreviated version of the PTGI with 10 items, selecting those with the highest factorial loading in each of the five original dimensions. Thus, was born the PTGI-SF, a brief tool that has demonstrated validity and reliability in various international contexts, such as the United States, Pakistan, Malaysia, Italy, Portugal, and Chile (Kaler et al., 2011; Qandeel et al., 2014; Prati and Pietrantoni, 2014; Lamela et al., 2014; García and Wlodarczyk, 2016). Although some studies have proposed a unifactorial structure (Kaur et al., 2017), the evidence predominantly supports its five-factor model.

Despite its acceptance, various studies have attempted to explain the factorial variability of the PTGI, attributing it to cultural, religious, and contextual differences according to the sample and country of application (Garrido-Hernansaiz et al., 2017; Tomita et al., 2016). It has also been observed that high correlations between factors may indicate substantial overlap among dimensions and raise questions regarding the effective dimensionality of the construct (Sears et al., 2003; Osei-Bonsu et al., 2012). Furthermore, the validity of early studies relying on principal component analysis has been questioned, given that this technique tends to overestimate the number of factors and does not adequately model latent structures (Lloret-Segura et al., 2014; Watkins, 2018). In this context, Exploratory Structural Equation Modeling (ESEM) has been recommended as a more appropriate analytic approach, as it integrates advantages of exploratory and confirmatory factor analysis while allowing non-zero cross-loadings and providing a more realistic representation of complex psychological constructs (Marsh et al., 2009; Morin et al., 2013).

Nevertheless, the psychometric properties of the Chilean Spanish adaptation of the PTGI-SF validated by García and Wlodarczyk (2016) have not yet been examined using contemporary latent variable modeling approaches. Specifically, evidence regarding its internal structure, reliability, and relations with external criteria remains limited in populations exposed to large-scale natural disasters. Consequently, the objective of the present study was to evaluate the psychometric properties of the PTGI-SF in a Chilean population exposed to the February 27, 2010, earthquake, focusing on its latent factorial structure through Exploratory Structural Equation Modeling (ESEM), internal consistency, and criterion-related validity. Adequate psychometric validation is essential to ensure the accuracy of assessment and to support the appropriate use of psychological instruments in post-disaster intervention and research contexts (Cadell et al., 2015).

Based on previous international evidence and methodological considerations, the following hypotheses were formulated:

H1. Competing latent structures (including the original five-factor and alternative reduced-factor solutions) are expected to show that a multidimensional model provides the best representation of PTGI-SF responses in disaster-exposed Chilean communities, with interpretable factors and adequate fit under ESEM.
H2. The scale is expected to demonstrate high internal consistency across its dimensions or subscales. Specifically, each subscale is anticipated to yield a McDonald's Omega coefficient (ω) greater than 0.70, with narrow confidence intervals indicating precision in estimation and satisfactory reliability of the scores.
H3. The scale is expected to show evidence of criterion-related validity. In particular, scale scores are expected to exhibit significant associations, in the theoretically expected direction, with relevant external criterion variables (e.g., other instruments assessing similar constructs or known-group differences), supporting the instrument's practical utility.
H4. The factorial structure of the PTGI-SF is expected to be equivalent across the communities analyzed. From an inferential perspective, at least configural and metric measurement invariance (MI) is anticipated under multigroup CFA with WLSMV estimation, supporting comparability of the factor configuration and factor loadings across groups. Additionally, as a supplementary descriptive indicator of similarity in loading patterns, Tucker's congruence coefficients will be reported; values ≥0.95 will be interpreted as high structural similarity.

## 2 Materials and method

The validation framework followed the Standards for Educational and Psychological Testing (American Educational Research Association, American Psychological Association, and National Council on Measurement in Education, 2014), including evidence based on internal structure (ESEM), reliability, and relations to other variables (criterion and concurrent validity).

### 2.1 Participants

This sample represents adults residing in three Chilean coastal and inland communities affected by the 2010 earthquake. The population is characterized by socioeconomic vulnerability, low formal education, and occupations linked to informal labor, fishing, and domestic work. Thus, generalization should be restricted to disaster-exposed populations with similar cultural and socioeconomic profiles.

A total of 578 adults from three Chilean communes participated: Pelluhue (*n* = 179), Cauquenes (*n* = 202), and Tongoy (*n* = 197). Participant selection was conducted through mixed, two-stage, and stratified sampling. In the first stage, blocks and dwellings were randomly selected; in the second stage, the head of household was interviewed.

The target sample size was defined *a priori* based on analytic considerations relevant to latent variable modeling rather than on heuristic rules of thumb. Specifically, recommendations for structural equation modeling were considered in relation to the number of observed variables (*k* = 10), the expected level of communalities (anticipated to be moderate-to-high based on prior PTGI-SF studies), and the complexity of the factorial solution to be estimated. Simulation and analytic work have shown that, under conditions of high communalities and moderate model complexity, stable factor recovery and accurate parameter estimation can be achieved with moderate sample sizes (MacCallum et al., 1999; Marsh et al., 2009). Under these conditions, a total sample of *N* = 578 was deemed sufficient to ensure adequate statistical power and robust estimation of the ESEM parameters.

The sample was composed predominantly of women (68.0%). Regarding employment status, 43.6% of participants were engaged in unpaid domestic work, while 9.9% worked in commerce and 6.1% in artisanal fishing activities. Concerning educational level, 68.9% had not completed secondary education, with complete secondary education being the most frequent level (31.5%), followed by incomplete primary education (22.5%).

In relation to marital status, a high prevalence of individuals without stable partners was observed: 23.2% separated, 22.3% widowed, and 17.0% single, while only 15.2% were married. The average household size was 3.5 people, with 69.4% of households composed of two to four members. These sociodemographic characteristics evidence the socioeconomic vulnerability profile of the coastal communities studied (see Table 1).

**Table 1:** Sociodemographic characteristics and disaster exposure of participants (*N* = 578).

Characteristic	Category/Description	*n*	%
Place of origin	Pelluhue	179	31.0
	Cauquenes	202	34.9
	Tongoy	197	34.1
Gender	Women	393	68.0
	Men	185	32.0
Age group	55–65 years	134	23.2
	Other age ranges	444	76.8
Employment status	Unemployed	332	57.4
	Working without a contract	152	26.3
	Other situations	94	16.3
Monthly household income	< $400,000 CLP	481	83.3
	≥$400,000 CLP	97	16.7
Material impact of disaster	Total loss of housing	100	17.3
	Severe damage to housing	78	13.5
	No severe damage or total loss	400	69.2
Educational level	Completed secondary	182	31.5
	Incomplete primary	130	22.5
	Completed primary	86	14.9
	Incomplete secondary	85	14.7
	Technical/Higher education	41	7.1
	University	37	6.4
	No formal education	16	2.8

Percentages are based on the total sample (N = 578) and may not sum to 100% within each characteristic due to rounding. Monthly household income is reported in Chilean pesos (CLP). “Material impact of disaster” refers to self-reported housing damage/loss attributable to the February 27, 2010, earthquake.

This study was conducted in accordance with the Declaration of Helsinki and was approved by the Bioethics Committee of the University of Talca (approval number: 00117, dated June 27, 2012). All participants provided written informed consent following the guidelines of the Council for International Organizations of Medical Sciences (2002), which stipulate the research objectives, reasons for their selection, the voluntary nature of participation, freedom to withdraw, duration of participation, and the benefits and sponsors of the research. Participants were informed that some questions might evoke memories of the February 27, 2010 earthquake, and that the interview could be suspended if distress prevented them from continuing.

### 2.2 Study design

This was a non-experimental, cross-sectional, psychometric-correlational study (Montero and León, 2002). No variables were manipulated; rather, relationships between constructs were observed in their natural context. The evaluation of causal models was based on statistical control of variables, not on experimental manipulation (Ruiz et al., 2010).

### 2.3 Instruments

Post-Traumatic Growth Inventory–Short Form (PTGI-SF). Developed by Cann et al. (2010) as an abbreviated version of the original PTGI (Tedeschi and Calhoun, 1996), this instrument contains 10 items originally intended to represent five content domains: New Possibilities (items 4 and 7), Relating to Others (items 5 and 10), Personal Strength (items 6 and 9), Appreciation of Life (items 1 and 2), and Spiritual Change (items 3 and 8). Items are rated on a 6-point scale reflecting the degree of positive change experienced as a result of the traumatic event (0 = *I did not experience this change as a result of my crisis*, 5 = *I experienced this change to a very great degree as a result of my crisis*). No standardized cut-off points have been established; higher scores indicate greater perceived post-traumatic growth, and values in the upper range (e.g., 3–5) are sometimes used descriptively as moderate-to-high growth in applied research contexts (Esparza-Baigorri et al., 2016).

Short Post-traumatic Stress Disorder Rating Interview (SPRINT-E). Post-Traumatic Stress Disorder (PTSD) symptoms were evaluated using the Short Post-traumatic Stress Disorder Rating Interview (SPRINT-E), developed by Norris et al. (2008) and adapted to the Chilean context by Leiva-Bianchi and Gallardo (2013). The instrument is composed of 12 items; items 1 through 11 are answered on an intensity Likert scale ranging from 0 (not at all) to 4 (extremely), while item 12 evaluates suicidal ideation dichotomously (0 = no ideation; 1 = with ideation). An item is considered indicative of intense symptomatology when scored with a value equal to or greater than 3 (or 1 in the case of item 12). According to the criteria established by Norris et al. (2008), the presence of three or more intense symptoms suggests a probable case of PTSD, with low probability of false positives when seven intense symptoms are exceeded.

### 2.4 Procedure

Data collection was conducted through face-to-face interviews in homes, directed to the head of the household, or to the second responsible adult in their absence. All participants were over 18 years old. The surveys were administered by trained interviewers, with experience at the National Institute of Statistics of Chile (INE) or training in psychology. The study objective was presented, informed consent was obtained, and the instruments were applied in a structured manner. The study is part of broader research on the psychosocial consequences of the February 27, 2010, earthquake in Chile.

### 2.5 Data analysis

Sample adequacy was first examined using the Kaiser–Meyer–Olkin index (KMO ≥0.80) and Bartlett's test of sphericity (*p* < 0.05) to verify that the data were suitable for factor extraction. Content validity coefficients were not estimated because the PTGI-SF is an established instrument and no item development or content adaptation was conducted in this study. Internal structure validity evidence was subsequently evaluated through Exploratory Structural Equation Modeling (ESEM), which allowed examination of the internal structure of the PTGI-SF while accounting for potential cross-loadings between items. Exploratory Structural Equation Modeling (ESEM; Asparouhov and Muthén, 2009; Marsh et al., 2009) was performed to define the factorial structure of the instrument, using established model fit criteria: RMSEA ≤ 0.05, CFI ≥0.85, and TLI ≥0.90. Factor loadings ≥0.40 were considered salient, and items with cross-loadings greater than.30 or with differences between primary and secondary loadings smaller than 0.20 were carefully examined, following current psychometric recommendations (Ferrando and Lorenzo-Seva, 2013; Howard, 2016; Lloret-Segura et al., 2014; Williams et al., 2010). All structural analyses were conducted using Mplus statistical software (Muthen & Muthen, Los Angeles, CA, USA).

The reliability of the PTGI-SF was estimated using McDonald's ordinal omega (Ω ≥ 0.70), which is generally considered more appropriate than Cronbach's alpha under conditions of congeneric measurement and unequal factor loadings. However, omega estimates are sensitive to model specification, dimensionality, and scale length, and may vary depending on the underlying factor structure and item characteristics. Therefore, omega coefficients were interpreted cautiously and in conjunction with the latent structure identified through ESEM (Finch et al., 2016; Goodboy and Martin, 2020; Ventura-León and Caycho-Rodríguez, 2017). Reliability was examined for both the PTGI-SF total score and the latent dimensions derived from the ESEM solution (Appreciation of Life, Spiritual Change, and Self-Perception). Confidence intervals for Ω were obtained using non-parametric bootstrap resampling (1,000 iterations), reporting 95% percentile-based confidence intervals.

Item-level factorial complexity was quantified using Hofmann's (1978) complexity coefficient, which summarizes how strongly an item loads on multiple factors in the rotated solution. Specifically, the coefficient is computed from the item's squared loadings across all extracted factors and reflects the extent to which the item departs from simple structure (higher values indicating greater cross-loading complexity). Complexity coefficients were obtained using the facomplex procedure (Pettersson and Turkheimer, 2010) as implemented in the facomplex package (Merino-Soto, 2025).

Criterion-related validity was examined by contrasting PTGI-SF scores with levels of post-traumatic stress symptoms measured using the SPRINT-E scale, applying as clinical criterion the presence of seven or more intense symptoms. Discriminative capacity was evaluated using Receiver Operating Characteristic (ROC) curve analysis. Area under the curve (AUC) values between 0.70 and 0.79 were interpreted as acceptable, values between 0.80 and 0.89 as good, and values above 0.90 as excellent, following established guidelines (Safari et al., 2016; Vizcaíno-Salazar, 2017). ROC analyses were conducted using MedCalc software (version 20.010) (MedCalc Software Ltd, Ostend, Belgium), with confidence intervals estimated via the BCa bootstrap method (1,000 resamples; random seed = 978) to ensure stability and reproducibility of results. The optimal cut-off point for the PTGI-SF was determined using the Youden index, prioritizing solutions that maximized sensitivity (ideally above 60%) while maintaining adequate specificity to reduce false-positive classifications. Concurrent validity was further evaluated through Pearson correlation analyses between PTGI-SF scores and the total score of the SPRINT-E, with the aim of empirically examining the association between post-traumatic growth and post- traumatic stress symptomatology.

Finally, the degree of similarity between the factorial loading configurations obtained for the three local models (Cauquenes, Pelluhue, and Tongoy) was initially examined using Tucker's congruence coefficient (CC; Tucker, 1969) and the salient variable similarity index (S; Cattell, 1960). These indices provide descriptive information regarding the similarity of factor loading patterns across groups. CC values greater than 0.95 were interpreted as indicating a high degree of factorial similarity (Lorenzo-Seva and ten Berge, 2006), while S values close to 1 suggested similarity in the salient loading pattern. However, because congruence indices do not constitute a formal statistical test of measurement equivalence, factorial similarity was subsequently evaluated through a measurement invariance (MI) framework using multigroup confirmatory factor analysis. Following current psychometric recommendations, configural, metric, and scalar invariance were tested to assess whether the PTGI-SF operated equivalently across communities, allowing for increasingly restrictive comparisons across groups (Cheung and Rensvold, 2002; Putnick and Bornstein, 2016). This combined approach ensured that descriptive evidence of factorial similarity was complemented by formal inferential tests of measurement invariance prior to substantive cross-group interpretations.

## 3 Results

### 3.1 Internal structure validity evidence and reliability

Table 2 presents the fit indices and reliability estimates obtained for the PTGI-SF ESEM models in the Cauquenes, Pelluhue, and Tongoy samples. Sampling adequacy was supported across localities, with KMO values above 0.80; Bartlett's tests of sphericity were significant (ps < 0.01), indicating that the correlation matrices were suitable for factor extraction. Model fit differed substantially across communities. The Tongoy model showed acceptable fit (χ^2^_(18)_ = 41.12, RMSEA = 0.08, CFI/TLI = 0.99), whereas the Pelluhue model showed poor fit despite high incremental indices (χ^2^_(18)_ = 81.45, RMSEA = 0.14, CFI = 0.98, TLI = 0.95). The Cauquenes model showed clearly unacceptable fit (χ^2^_(18)_ = 193.11, RMSEA = 0.22, CFI = 0.92, TLI = 0.80), suggesting notable model misspecification in this community. The discrepancy between high CFI/TLI and elevated RMSEA is consistent with known behavior of fit indices in small-df ordinal models, where RMSEA may be inflated even when incremental indices remain high. Because of item-level missingness, effective sample sizes for some analyses may vary slightly by locality (see Table 2).

**Table 2:** Fit indices and reliability of the factorial model of PTGI-SF Cauquenes, PTGI Pelluhue, and PTGI Tongoy.

Localidad	*n*	Factores	KMO	χ^2^ (df)	% var	SRMR	RMSEA	CFI	TLI	Ω total (IC95%)	Ω AV (IC95%)	Ω CE (IC95%)	Ω AP (IC95%)
Cauquenes	202	3	0.80	193.11^*^ (18)	76.7	0.05	0.22	0.92	0.80	0.91 (0.88–0.94)	0.875 (0.784, 0.937)	0.947 (0.922, 0.965)	0.937 (0.911, 0.960)
Pelluhue	179	3	0.87	81.45^*^ (18)	77.1	0.03	0.14	0.98	0.95	0.91 (0.89–0.93)	0.782 (0.612, 0.880)	0.889 (0.835, 0.929)	0.857 (0.790, 0.915)
Tongoy	197	3	0.91	41.12^*^ (18)	85.5	0.01	0.08	0.99	0.99	0.95 (0.93–0.97)	0.860 (0.798, 0.910)	0.899 (0.865, 0.927)	0.940 (0.917, 0.958)

KMO, Kaiser–Meyer–Olkin measure of sampling adequacy; χ^2^(df), model chi-square test for the ESEM solution (degrees of freedom in parentheses); % var, percentage of variance explained; SRMR, standardized root mean square residual; RMSEA, root mean square error of approximation; CFI, comparative fit index; TLI, Tucker–Lewis index. Ω, McDonald's ordinal omega; values in brackets indicate 95% bootstrap confidence intervals (1,000 resamples). AV, Appreciation of Life; CE, Spiritual Change; AP, Self-Perception. Bartlett's tests of sphericity were significant in all samples (ps < 0.01).

^*^p < 0.01.

**Figure 1:**
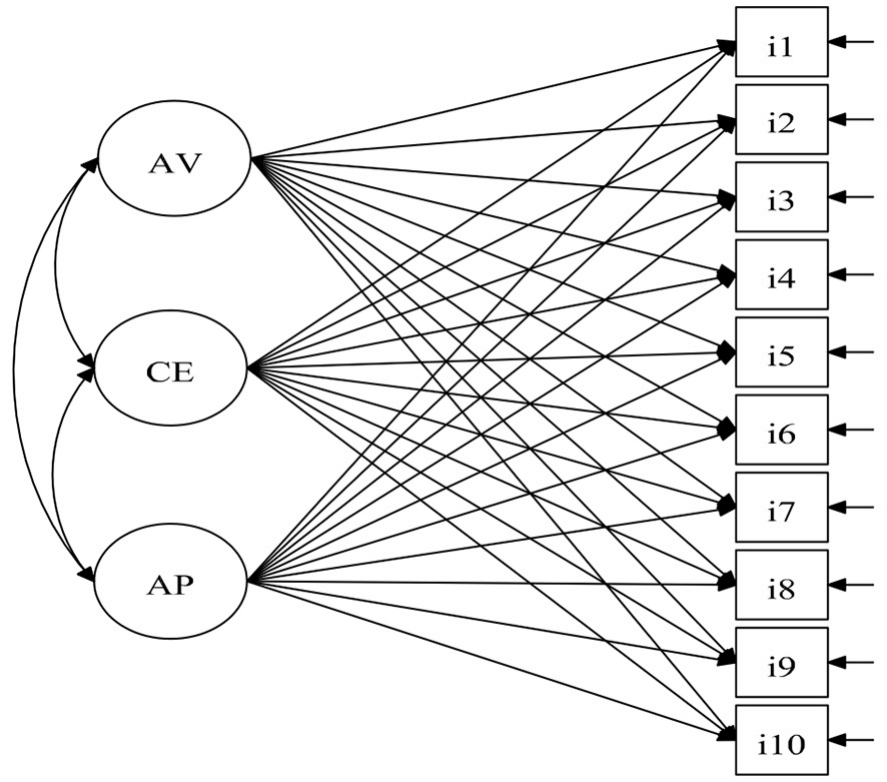
Path diagram of the three-factor Exploratory Structural Equation Modeling (ESEM) solution for the Post-Traumatic Growth Inventory–Short Form (PTGI-SF). Ellipses represent the three latent factors: Appreciation of Life (AV), Spiritual Change (CE), and Self-Perception (AP). Rectangles represent the 10 observed items (i1–i10). Single-headed arrows from latent factors to observed items denote factor loadings (including cross-loadings freely estimated under ESEM). Double-headed arrows between latent factors represent interfactor correlations. Small arrows pointing to each observed item indicate residual (unique) variances.

Figure 1 graphically illustrates the factorial structure of the ESEM solution for the PTGI-SF across the three communities, composed of Appreciation of Life (AV), Spiritual Change (CE), and Self-Perception (AP).

Table 3 details the factor loadings and standard errors for each item across the three local models. Loadings were generally high on their primary factors, particularly for items defining Appreciation of Life and Self-Perception. Some items presented minor or negative cross-loadings, which were considered in the overall evaluation of the solution. Factor complexity was computed using the “facomplex” procedure (Merino-Soto, 2025). Items showed moderate complexity (range 1.18–1.42), indicating that the ESEM solution captured the expected cross-loading structure of a multidimensional construct.

**Table 3:** Factor loadings and standard errors of the ESEM models of the PTGI-SF by locality (AV/CE/AP).

Item	Item content	Cauquenes AV	Pelluhue AV	Tongoy AV	Cauquenes CE	Pelluhue CE	Tongoy CE	Cauquenes AP	Pelluhue AP	Tongoy AP	Factorial complexity (Cauq/Pell/Tong)
01	Cambié escala de valores/prioridades	0.881^*^ (0.087)	0.809^*^ (0.103)	0.936^*^ (0.042)	−0.128 (0.083)	−0.034 (0.034)	0.006 (0.040)	0.003 (0.007)	−0.073 (0.066)	−0.060 (0.033)	2.96/1.15/1.93
02	Aprecio más el valor de mi vida	0.723^*^ (0.075)	0.570^*^ (0.122)	0.574^*^ (0.091)	0.011^*^ (0.003)	0.121 (0.086)	0.045 (0.060)	0.275^*^ (0.068)	0.330^*^ (0.094)	0.356^*^ (0.114)	2.84/1.29/2.88
03	Soy más espiritual/crecí espiritualmente	−0.011 (0.010)	0.066^*^ (0.033)	0.021 (0.063)	0.927^*^ (0.055)	0.966^*^ (0.044)	0.976 (0.124)	−0.094 (0.064)	−0.061 (0.026)	−0.014 (0.068)	1.26/2.99/1.77
04	Construí un nuevo rumbo/caminos de vida	0.167^*^ (0.083)	0.020 (0.063)	0.509^*^ (0.093)	0.296^*^ (0.090)	0.356^*^ (0.099)	0.227 (0.088)	0.275^*^ (0.079)	0.430^*^ (0.099)	0.163 (0.104)	1.22/2.68/2.03
05	Mayor cercanía/proximidad con los demás	0.159^*^ (0.065)	0.036 (0.053)	0.398^*^ (0.077)	0.467^*^ (0.077)	0.377^*^ (0.114)	0.122 (0.073)	0.275^*^ (0.065)	0.506^*^ (0.110)	0.461^*^ (0.105)	1.33/2.07/2.54
06	Me las arreglo mejor en momentos difíciles	−0.111 (0.136)	0.135^*^ (0.055)	−0.078 (0.031)	0.066 (0.058)	0.092 (0.086)	0.005 (0.041)	0.828^*^ (0.092)	0.659^*^ (0.088)	0.993^*^ (0.045)	2.58/1.82/2.72
07	Capaz de hacer cosas mejores en mi vida	−0.228 (0.149)	0.131^*^ (0.067)	0.013 (0.066)	−0.014 (0.009)	0.234^*^ (0.083)	0.003 (0.051)	0.997^*^ (0.084)	0.610^*^ (0.088)	0.898^*^ (0.080)	1.60/1.01/2.66
08	Tengo más fe religiosa	−0.263^*^ (0.074)	−0.102^*^ (0.038)	−0.052 (0.049)	1.051^*^ (0.065)	0.745^*^ (0.118)	0.906 (0.128)	0.003 (0.003)	0.173 (0.122)	0.004 (0.090)	1.38/2.79/1.00
09	Descubrí que soy más fuerte de lo que pensaba	0.001 (0.010)	0.028 (0.041)	0.215^*^ (0.074)	0.420^*^ (0.060)	−0.071 (0.052)	0.305 (0.072)	0.511^*^ (0.060)	0.894^*^ (0.053)	0.454^*^ (0.078)	1.03/1.89/2.14
10	Aprendí lo maravillosas/extraordinarias que son las personas	0.071 (0.066)	−0.101^*^ (0.035)	0.216^*^ (0.069)	0.371^*^ (0.068)	−0.015 (0.073)	0.103 (0.065)	0.493^*^ (0.063)	0.857^*^ (0.067)	0.660^*^ (0.086)	1.66/1.16/2.53

Values are standardized ESEM factor loadings; standard errors are shown in parentheses. For each locality, factors were labeled according to the dominant loading pattern: Appreciation of Life (AV; items 1–2), Spiritual Change (CE; items 3, 4, 5, and 8), and Self-Perception (AP; items 6, 7, 9, and 10). Because factor ordering may vary across localities under ESEM rotation, factor labels reflect item content rather than numerical order. Cross-loadings are freely estimated (ESEM) and therefore are reported in full.

^*^p < 0.01. Loadings slightly above 1.00 may occur in rotated ESEM solutions under high factor correlations or near-boundary residual variances and should be interpreted cautiously in conjunction with the full model diagnostics.

Latent interfactor correlations were estimated from the ESEM model to further examine the internal structure of the PTGI-SF. Table 4 presents the interfactor correlations for each locality (Cauquenes, Pelluhue, and Tongoy). Correlations were positive and statistically significant across models, with magnitudes ranging from low to high, supporting the conceptualization of post-traumatic growth as a multidimensional construct composed of related but distinguishable dimensions.

**Table 4:** Interfactor correlations (latent variables) derived from the ESEM model by locality.

Factor	Appreciation of life	Spiritual change	Self-perception
Panel A. Cauquenes			
Appreciation of life	–	0.587^*^	0.527^*^
Spiritual change	0.587^*^	–	0.633^*^
Self-perception	0.527^*^	0.633^*^	–
Panel B. Pelluhue			
Appreciation of life	–	0.460^*^	0.330^*^
Spiritual change	0.460^*^	–	0.700^*^
Self-perception	0.330^*^	0.700^*^	–
Panel C. Tongoy			
Appreciation of life	–	0.268^*^	0.773^*^
Spiritual change	0.268^*^	–	0.221^*^
Self-perception	0.773^*^	0.221^*^	–

Values correspond to latent factor correlations estimated from the ESEM model using Geomin rotation.

^*^p < 0.05.

Regarding reliability, McDonald's ordinal Omega was high for the total score across localities (Ω = 0.91–0.95; Table 2). Consistent with a multidimensional solution, Omega estimates were also examined at the factor level. Subscale reliability was acceptable-to-high across localities (Table 2), with the lowest estimates observed for Appreciation of Life in Pelluhue and the highest estimates observed for Spiritual Change and Self-Perception across groups.

### 3.2 Criterion validity

A ROC curve analysis was performed to evaluate the capacity of the PTGI-SF to discriminate between participants with and without severe post-traumatic symptomatology, defined by the presence of 7 or more intense symptoms according to the SPRINT-E. The total sample was 578 participants, of which 28 (4.8%) were classified as positive cases of severe PTSD.

**Figure 2:**
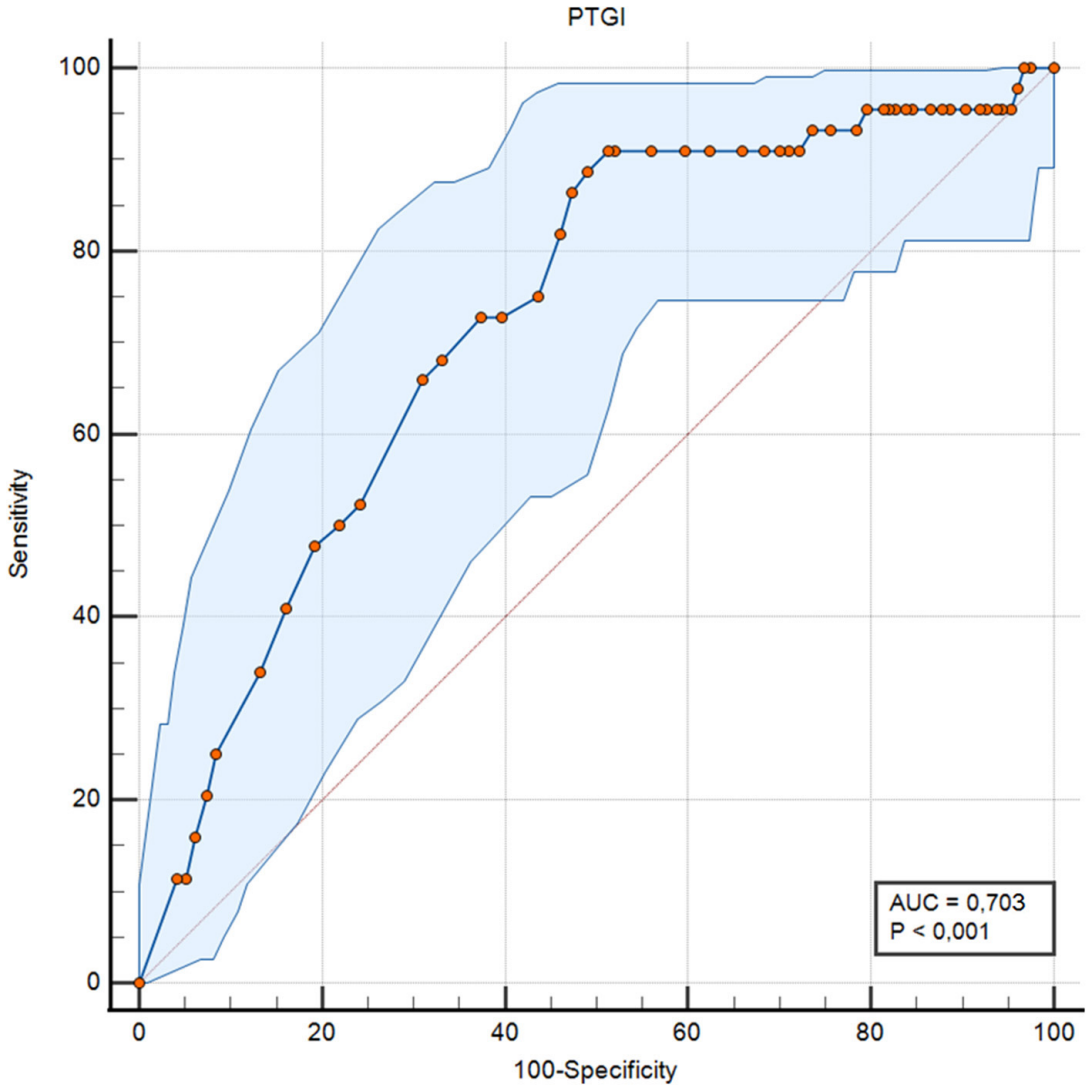
Receiver Operating Characteristic (ROC) curve for the PTGI-SF total score as a predictor of severe post-traumatic stress symptomatology (≥7 intense symptoms on the SPRINT-E). The solid line represents the empirical ROC curve, the shaded area indicates the 95% confidence band (BCa bootstrap, 1,000 resamples), and the diagonal dashed line represents the reference line of no discrimination. AUC, area under the curve.

The ROC analysis showed moderate discriminative capacity (AUC = 0.703; 95% CI: 0.630–0.770) for identifying severe post-traumatic stress symptomatology (Figure 2). High sensitivity (90.91%) coupled with limited specificity (48.69%) suggests restricted utility for screening purposes and clear limitations for clinical decision-making.

**Figure 3:**
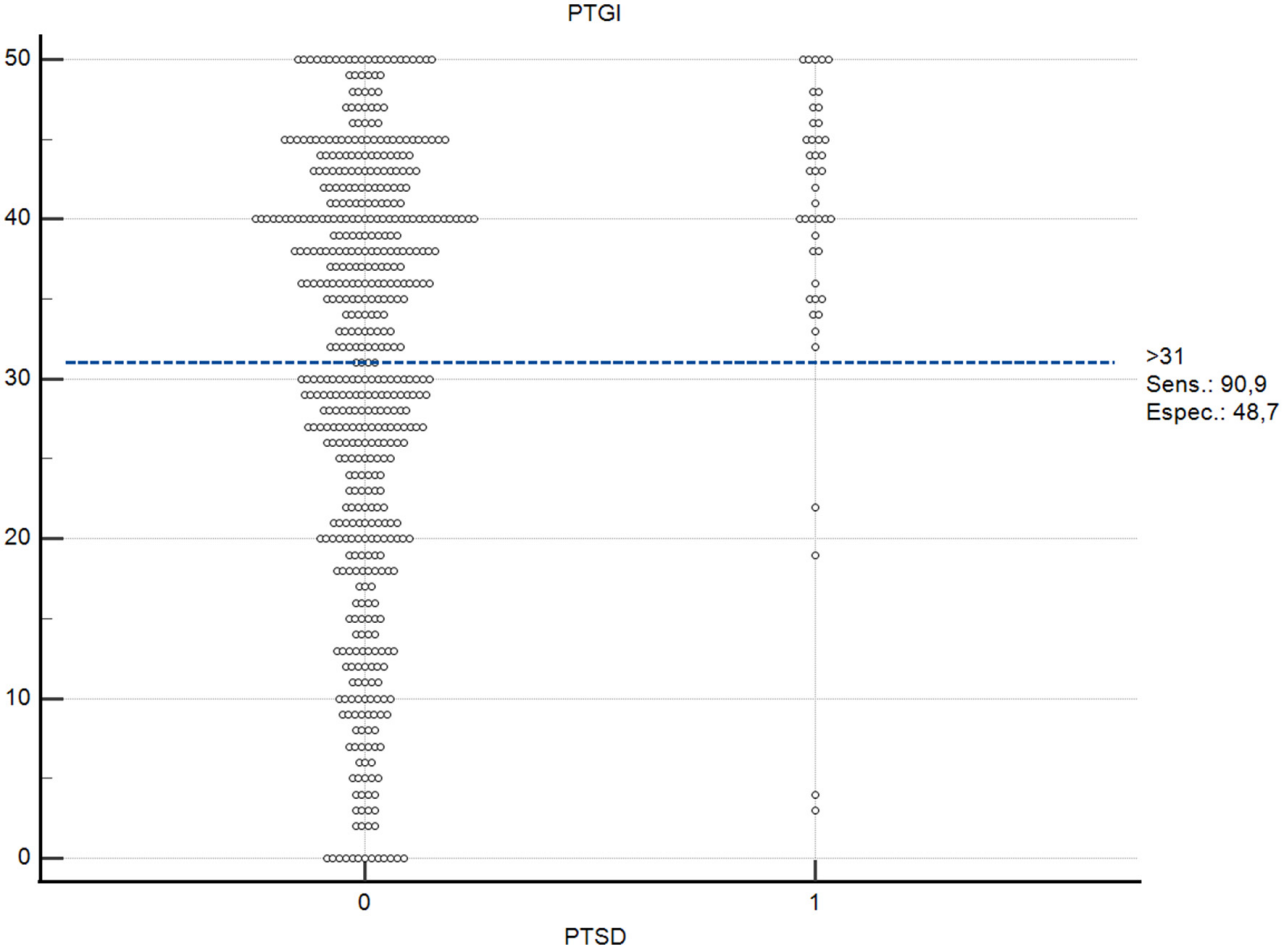
Interactive dot plot displaying the distribution of PTGI-SF total scores by PTSD classification (0 = non-case; 1 = severe PTSD case, defined as ≥7 intense symptoms on the SPRINT-E). The horizontal dashed line indicates the optimal cut-off point (>31) determined by the Youden index, with corresponding sensitivity (90.9%) and specificity (48.7%).

Additionally, the distribution graph of cases as a function of PTGI-SF score showed a greater concentration of individuals with severe PTSD above the proposed cut-off point, supporting the pattern observed in the ROC analysis (Figure 3).

**Figure 4:**
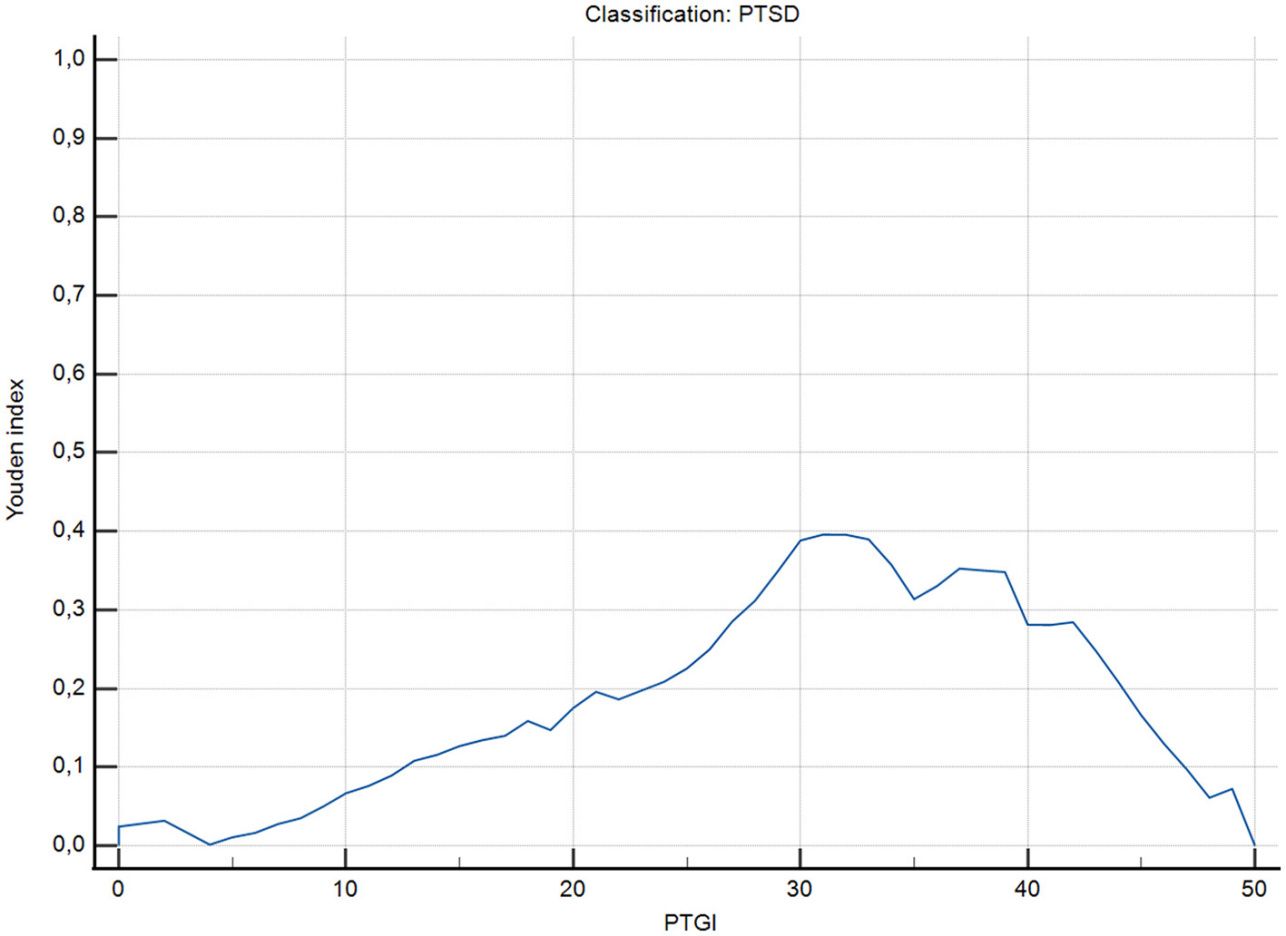
Youden index as a function of PTGI-SF total score for the classification of severe PTSD (≥7 intense symptoms on the SPRINT-E). The peak of the curve indicates the optimal cut-off point that maximizes the combination of sensitivity and specificity.

Given that the PTGI-SF measures post-traumatic growth (a positive construct), scores were not reverse-coded. Importantly, discriminative performance in this context should not be interpreted as implying an inverse relation between PTG and PTSD. Rather, the ROC results and the concurrent validity findings below are consistent with the documented possibility of co-occurrence between growth and distress following trauma (Tedeschi and Calhoun, 2004; Shakespeare-Finch and Lurie-Beck, 2014) (Figure 4).

### 3.3 Concurrent validity

Concurrent validity was evaluated through Pearson correlations between PTGI-SF total scores and SPRINT-E total scores. A positive and statistically significant correlation was observed (*r* = 0.288, *p* < 0.001), indicating a modest association between levels of post-traumatic growth and the severity of post-traumatic stress symptomatology.

### 3.4 Factorial similarity and measurement invariance across community contexts

As an initial descriptive approach, Tucker's congruence coefficients were computed to examine similarity in factor loading patterns across the three community contexts. Congruence coefficients provide an index of structural similarity but do not constitute a formal statistical test of measurement equivalence. Results indicated moderate similarity between Cauquenes and Pelluhue (CC = 0.89) and lower similarity for comparisons involving Tongoy (CC = 0.81), suggesting a broadly shared latent configuration with context-specific variations.

Given the descriptive nature of congruence coefficients, factorial similarity was further examined through formal tests of measurement invariance using multigroup CFA with WLSMV estimation. Configural invariance showed acceptable fit, indicating that the same three-factor structure was supported across the three communities (CFI = 0.94, TLI = 0.96), despite elevated RMSEA values, a pattern frequently observed in ordinal CFA models. These invariance results should be interpreted considering the locality-specific misfit observed in the ESEM models (particularly in Cauquenes), which may affect the stability of multigroup solutions. Metric invariance was subsequently supported, as constraining factor loadings across groups resulted in improved incremental fit indices (CFI = 0.95; TLI = 0.96) and a reduction in RMSEA (ΔCFI = +0.01; ΔRMSEA = −0.01), remaining within recommended thresholds. In contrast, scalar invariance could not be established due to inadmissible solutions in the Pelluhue group, characterized by a non-positive definite latent covariance matrix and correlations exceeding admissible bounds among latent factors, indicating insufficient empirical separation between dimensions in this context.

## 4 Discussion

The present study evaluated the psychometric properties of the PTGI-SF in 578 Chilean individuals exposed to the February 27th earthquake, providing differentiated support for the proposed hypotheses. ESEM analyses supported a three-factor structure comprising Appreciation of Life, Spiritual Change, and Self-Perception, diverging from the original five-factor model (H1 supported). Reliability evidence was strong for both the total score and the derived dimensions across localities (H2 supported). Criterion-related validity was acceptable but limited in specificity (AUC = 0.703; H3 supported with practical constraints), and concurrent validity showed a modest positive association between PTGI-SF and PTSD symptom severity (*r* = 0.288, *p* < 0.001), consistent with the possibility that growth and distress may co-occur following trauma (Tedeschi and Calhoun, 2004; Shakespeare-Finch and Lurie-Beck, 2014). Finally, equivalence across communities (H4) was only partially supported: configural and metric invariance were established, whereas scalar invariance could not be supported due to inadmissible solutions, and congruence coefficients suggested only moderate-to-low similarity across some locality comparisons.

The three-factor structure identified is consistent with prior evidence showing factorial variability of the PTGI-SF across cultural contexts and trauma types. Studies with diverse populations, including breast cancer patients (Porro et al., 2022), military personnel (Kaur et al., 2017), and COVID-19 affected individuals (Azman et al., 2023), have documented meaningful variation in how post-traumatic growth is structured and expressed. While the original five-factor model often emerges, these studies also suggest that item functioning and factor differentiation may depend on sociocultural context and event characteristics, reinforcing the need for localized validation when interpreting and using the PTGI-SF (Lamela et al., 2014).

The poor fit observed in Cauquenes (RMSEA = 0.22) requires cautious interpretation. This value exceeds acceptable thresholds and suggests substantive model misspecification in this locality. Plausible explanations include: (a) heterogeneity in trauma exposure and recovery conditions in inland vs. coastal communities; (b) unmeasured socioeconomic or contextual differences influencing response patterns; (c) the presence of latent subgroups with distinct PTG profiles; or (d) differential disaster impact related to geographic proximity and recovery resources. Accordingly, generalization to this locality should be conservative, and future work should explore alternative specifications and/or more flexible modeling approaches to better represent PTG in inland disaster-affected contexts.

The ROC analysis yielded moderate discriminative capacity for identifying severe PTSD symptomatology (AUC = 0.703). Although sensitivity was high (90.91%), specificity was limited (48.69%), indicating a substantial false-positive rate. This pattern supports a restricted screening role and is incompatible with diagnostic use. Conceptually, the limited specificity is also consistent with the instrument's scope: the PTGI-SF measures post-traumatic growth rather than the absence of psychopathology, and growth may emerge alongside distress in the post-trauma adaptation process (Tedeschi and Calhoun, 2004; Shakespeare-Finch and Lurie-Beck, 2014).

The ESEM results provide substantive support for three interpretable dimensions in this Chilean disaster-exposed sample. Appreciation of Life is clearly reflected by items 1 and 2, with consistently strong loadings across localities. Spiritual Change is represented by items 3, 4, 5, and 8, capturing a broader domain that integrates spiritual transformation together with meaning- and relationally oriented growth content, as indicated by the dominant loading pattern across the three communities (Włodarczyk et al., 2018; Ramos-Vera, 2022). Self-Perception is coherently represented by items 6, 7, 9, and 10, aligning with the notion of perceived personal strength and positive self-redefinition following adversity (Tedeschi et al., 2018).

Regarding equivalence across communities, the combination of descriptive congruence coefficients and formal MI tests provides a nuanced picture. Congruence suggested only moderate-to-low similarity across some comparisons (CC = 0.89 and 0.81), indicating that factor loading patterns are not fully interchangeable across all localities. Importantly, multigroup CFA supported configural and metric invariance, suggesting that the same three-factor configuration and the meaning of the latent constructs (in terms of loadings) were largely comparable across communities. However, scalar invariance could not be established due to inadmissible solutions, indicating that latent mean comparisons across communities are not supported and that context-specific response processes may be affecting item thresholds and/or factor separability.

The dimensional reduction from five to three factors raises questions about the cross-cultural universality of Tedeschi and Calhoun's (1996) original structure and may reflect: (1) culturally shaped integration of growth domains in Chilean disaster-exposed contexts; (2) trauma-type specificity in PTG manifestation; or (3) limitations of the short form in capturing finer-grained dimensional distinctions. This finding warrants further investigation, ideally incorporating mixed-methods work to clarify how disaster survivors in Chile conceptualize and report post-traumatic growth.

### 4.1 Limitations

Several limitations delimit interpretation. First, the cross-sectional design prevents evaluation of temporal stability and developmental trajectories of PTG. Longitudinal studies are needed to determine whether the three-factor structure and item functioning remain stable across recovery phases.

Second, model fit showed variability across localities, which suggests that the three-factor solution may be better represented in some contexts than in others. Although the three-factor structure was interpretable across communities, fit was more favorable in Tongoy (RMSEA = 0.08; CFI/TLI = 0.99), while RMSEA values were higher in Pelluhue (RMSEA = 0.14) and Cauquenes (RMSEA = 0.22; TLI = 0.80). This pattern indicates that contextual or sample-specific response characteristics may influence the degree to which the model captures the data. Therefore, interpretations regarding factorial validity should be made with attention to locality-specific fit, and conclusions about generalizability across communities should be stated cautiously.

Third, developmental trauma history and dissociative symptomatology were not assessed, despite their potential relevance for post-traumatic adaptation and growth trajectories (Miño-Reyes et al., 2024). Future research should incorporate these measures to better characterize survivor subgroups and PTG profiles.

Fourth, the study did not include cross-validation via CFA in an independent sample. Future studies should consider splitting the sample to perform exploratory ESEM in one subsample and confirmatory modeling in the other, as recommended by Brown (2015) and Marsh et al. (2014), to strengthen evidence for replicability.

Fifth, although formal measurement invariance analyses supported configural and metric invariance, scalar invariance could not be established due to inadmissible solutions. As a result, latent mean comparisons across communities should be interpreted cautiously, and future research should examine invariance using ESEM-based multigroup frameworks and/or complement MI with item-level approaches (e.g., DIF) to clarify whether non-invariance reflects threshold differences, local dependence, or reduced factor separability in specific contexts (Vandenberg and Lance, 2000; Putnick and Bornstein, 2016).

Sixth, convergent and discriminant validity evidence remains limited because additional PTG measures (e.g., full PTGI) and theoretically unrelated constructs were not included. Broader batteries would support a more complete nomological network evaluation (Campbell and Fiske, 1959).

Finally, face-to-face interviews may have introduced social desirability and context-driven response biases, particularly in post-disaster settings. These sources of bias may contribute to differential response patterns across communities and could partially account for the heterogeneity observed in model fit indices.

## 5 Conclusions

The PTGI-SF demonstrates acceptable psychometric performance in a Chilean post-earthquake sample, supporting a culturally specific three-factor structure with strong reliability. Evidence for relations to PTSD symptomatology was modest and consistent with co-occurrence of growth and distress, while screening utility was limited by low specificity. Cross-community equivalence was partially supported at configural and metric levels, but scalar non-invariance and inadmissible solutions preclude latent mean comparisons across localities. These findings underscore the importance of localized validation and cautious interpretation when applying the PTGI-SF across diverse disaster-affected communities.

## Data Availability

The raw data supporting the conclusions of this article will be made available by the authors, without undue reservation.
